# Analysis of Selected Methods Use for Calculation of the Coefficients of Adsorption Isotherms and Simplified Equations of Adsorption Dynamics with the Use of IZO Application

**DOI:** 10.3390/ma14154192

**Published:** 2021-07-27

**Authors:** Jacek Piekarski, Katarzyna Ignatowicz, Tomasz Dąbrowski

**Affiliations:** 1 Faculty of Civil Engineering, Environmental and Geodetic Sciences, Koszalin University of Technology, 75-453 Koszalin, Poland; jacek.piekarski@tu.koszalin.pl (J.P.); tomasz.dabrowski@tu.koszalin.pl (T.D.); 2 Faculty of Civil Engineering and Environmental Sciences, Białystok University of Technology, 15-351 Białystok, Poland

**Keywords:** adsorption process, adsorption isotherm, activated carbon, open-source application

## Abstract

The purpose of this paper is to present the IZO application that calculates and visualizes coefficients of adsorption isotherms according to Freundlich, Langmuir, and BET in a classic and linear system, in a simple communicative way. The application also calculates the working time of the adsorption bed based on the transformation of the mass balance equation, and according to the Zuchowicki, Zabieziński, Tichonow, and the Bohart-Adams equations. The laboratory tests of the adsorption process of leachate from a municipal landfill on selected active coals ORGANOSORB 10, DESOTEK, and BA-10, were conducted to check the program for accuracy. Results of tests confirm that the linearization method of the calculation of adsorption isotherms coefficients, used in the IZO application, gives sufficient accuracy and may be used as an alternative of, e.g., the nonlinear estimation method.

## 1. Introduction

There are several methods used to remove contaminants from water and wastewater. The adsorption process on activated carbons is one of the most effective methods. Due to the significant number of various impurities found in water and wastewater, the selection of activated carbon for use in the process of adsorption should be based on its adsorption properties. Adsorption properties depend on the chemical structure of the carbon surface and the number and type of surface groups [1,2]. The components adsorbed from the liquid solution form a sealed layer on the surface of the adsorbent. The equilibrium state of the adsorption process is defined as the equilibrium between the surface and volume solution compositions. The amount of adsorbed substances from the liquid phase is determined based on the difference in the composition of the volume and surface solution (excess adsorption) or based on the composition of the surface layer (real adsorption). The adsorption capacity is measured using static or dynamic methods [1,2,3]. The static method is based on determining the difference between the concentration of the adsorbed component in the initial solution and in the solution, which is in equilibrium with the adsorbent. Dynamic methods include, among others, complex chromatographic methods. The amount of individual organic substances adsorbed on activated carbon from aqueous solutions depends on the properties of an organic compound, such as molecular weight, dimension, geometric shape, type of functional groups, polarity, and solubility [1,2,3,4]. The adsorption capacity increases with the increase in molecular mass due to a more significant affinity of higher-mass molecules to the sorbent surface. However, the increase in the volume of the molecule of higher polarity and solubility decreases adsorption capacity because it limits the effective use of the adsorption capacity of activated carbon. The equations of adsorption isotherms, according to Freundlich, Langmuir, Brunauer, Emmett, and Teller (BET), are used, among others, to characterize the adsorption process on activated carbon [1,2,3,4,5,6,7,8].

The most common equation used for mathematical analysis of adsorption process on energetically heterogeneous surfaces and on microporous adsorbents is the experimental equation of adsorption isotherm, according to Freundlich, in the form *a* = *x_m_*∙*Cr*^1/*n*^. The value of the *x_m_* parameter varies in a wide range and shows the adsorption capacity of the sorbent at equilibrium concentration in the solution. A higher value of the *x_m_* coefficient corresponds to a higher adsorption capacity. On the other hand, the value of the *n* parameter allows assessing the adsorption intensity of a given substance on activated carbon from an aqueous solution. The more intensive process of adsorption, the higher value of *n*. The Freundlich isotherm cannot be applied at low concentrations, where the isotherm is linear (*n* = 1). It also cannot be used at high concentrations because, according to the Freundlich equation, the amount of adsorbed substance can increase indefinitely along with the equilibrium concentration increase, but in reality, adsorbent becomes saturated. Within the range of average values of equilibrium concentration, the Freundlich isotherm allows obtaining good results. Within this range, the course of Freundlich isotherm corresponds to Langmuir isotherm [1,2,5,6,9,10,11,12].

Langmuir’s theory assumes that the adsorbate can form the so-called monolayer of molecules interacting with adsorption sites on the adsorbent surface. The layer of adsorbate blocks adsorption forces and prevents the formation of subsequent layers. Langmuir’s theory applies the isotherm equation in the form *a* = *x_m_*∙[(*K_L_*∙*Cr*)/(1 + *K_L_*∙*Cr*)]. The adsorbate particles present in the aqueous solution hit the surface of the adsorbent. The chance of their adsorption increases with the available free space on the adsorbent. Adsorbed particles may also be desorbed. The phenomena of adsorption and desorption depend on the temperature and the value of adsorption energy. Simultaneously adsorbate particles hit more frequently against the adsorbent surface along with the increase in the equilibrium concentration. Moreover, increasing the number of adsorbed particles causes a decrease in the available surface. The assumptions of Langmuir’s theory also include no side interactions, no possibility of creating a multilayer, and constant adsorption energy (energetically homogeneous surface). At high equilibrium concentrations, the adsorbing surface reaches saturation, and then further increase in concentration does not increase the adsorption. In such a range of equilibrium concentrations, the adsorption isotherm is linear, parallel to the *y*-axis. The adsorption capacity is linearly proportional to the equilibrium concentration at low equilibrium concentrations [1,2,5,6,9,10,13,14,15].

In water and wastewater treatment, particles often form a multilayer system. Hence the Langmuir model loses its validity in such conditions. Stephan Brunauer, Paul Emmett, and Edward Teller developed the model (BET), considering the possibility of additional interaction between molecules. Therefore Langmuir isotherm is better for chemisorption. BET isotherm is better for physisorption on a non-microporous surface. The BET isotherm can be written as *a* = *x_m_*∙(*K_B_*∙*Cr*)/[(1 − *Cr*) (1 + (*K_B_* − 1) *Cr*)] [1,2,5,6,13,16,17,18,19,20].

Calculations of the duration of the adsorption process are considered on three levels. The first one concerns analyzing static factors influencing the equilibrium state and is expressed by the adsorption isotherm. The second level is the analysis of kinetic factors. The mathematical notation contains the equations of the kinetics of the adsorbate flow outside the adsorbent particle and the equations of the saturation kinetics of the adsorbent particles. The third level is the dynamics of adsorption. The notation uses the equations of statics and kinetics of adsorption and the equations of the mass balance of the adsorbent. The most general model describing the adsorption process in a batch system is the one based on the adsorbate concentration field’s equation in the adsorbent’s internal structure. In contrast, the dynamics of adsorption are considered by the time-spatial distribution of the adsorbate in the adsorbent bed [21].

**Figure 1:**
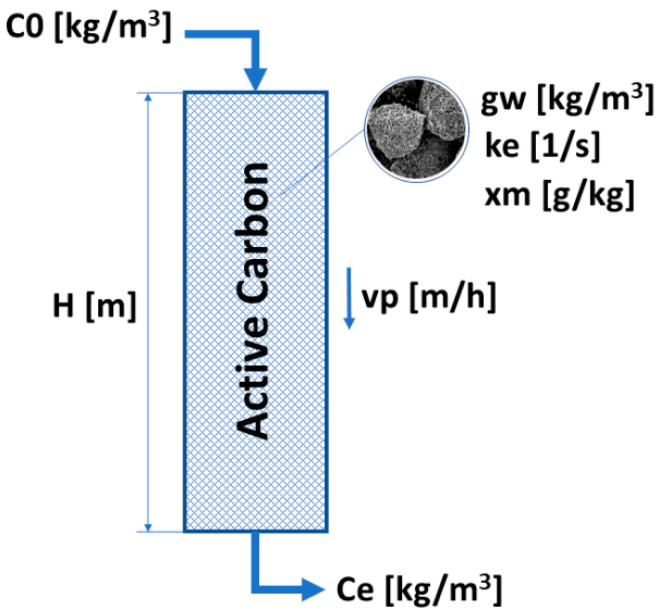
Simplified diagram of an adsorption bed.

When considering the calculation of the duration of the adsorption process on granular activated carbon, certain independent variable parameters are adopted. Parameters that concern the medium are: input concentration *C*_0_ [kg/m^3^], output concentration *Ce* [kg/m^3^] and flow velocity *vp* [m/h]. Parameters characterizing adsorbent are bed height *H* [m] and bulk density *gw* [kg/m^3^]. Additional independent variable parameters are external mass transfer coefficient *ke* [1/s] and adsorbate concentration in the adsorbent layer *xm* [g/kg] (Figure 1) [21].

During the adsorption process, the so-called adsorption front is formed. It decreases the value of *C*_0_ to *Ce*. Its height is much lower than the bed height. During the process, the front moves down the column. The adsorption process ends when the adsorption front reaches the end of the column, i.e., when *Ce* is exceeded in the outflowing solution.

Certain simplifying assumptions are adopted during the modeling of the adsorption process on granular activated carbon: the flowing medium is incompressible, the volume flow is constant over time, the bed is homogeneous, and it has the same porosity value in each cross-section. Moreover, the value of the mass transfer coefficient during the process is constant. Therefore, the adsorption capacity throughout the entire grain cross-section is the same. Transport of the adsorbate mass along the bed is carried out due to hydraulic flow diffusion [9,12,18,21,22,23,24,25].

The appropriate level of accuracy obtained in adsorption processes mostly depends on correct modeling and, as a result, the interpretation of adsorption isotherms. Complex mathematical computer applications are currently used for modeling, such as Statistica (https://www.statsoft.pl/ (accesed on 21 July 2021)), Matlab (https://www.mathworks.com/products/matlab.html (accesed on 21 July 2021)), TableCurve2D (http://www.sigmaplot.co.uk/products/tablecurve2d/tablecurve2d.php (accesed on 21 July 2021)), CurveExpert (https://www.curveexpert.net/ (accesed on 21 July 2021)), Originlab (https://www.originlab.com/ (accesed on 21 July 2021)), Eureqa (https://www.creativemachineslab.com/eureqa.html (accesed on 21 July 2021)), Proast (https://www.rivm.nl/en/proast (accesed on 21 July 2021)), DynaFit (http://www.biokin.com/dynafit/ (accesed on 21 July 2021)), SciDAVis (http://scidavis.sourceforge.net/ (accesed on 21 July 2021)), LAB Fit (http://www.labfit.net/ (accesed on 21 July 2021)). Moreover, software environments for statistical calculations and graphics such as R Projekt (https://www.r-project.org/ (accesed on 21 July 2021)) may be applied. However, these computer programs use numerical integration and require knowledge of programming or complicated operation. That is why they are not popular among researchers.

Currently, there is a lack of specialized computer programs that, in an accessible way, cover in detail the issues of calculating the duration of the adsorption process on a granular activated carbon bed, using the value of the adsorbate concentration in the adsorbent layer obtained from adsorption isotherms. Therefore, to solve that problem, the IZO computer application was developed. Moreover, the computational linearization method of adsorption isotherms implemented into the program was checked for accuracy by nonlinear estimation under laboratory conditions [22,26,27,28]. The laboratory tests of the adsorption process of leachate from a municipal landfill on selected active coals ORGANOSORB 10, DESOTEK, and BA-10, were conducted.

## 2. Materials and Methods

### 2.1. Characteristics of IZO Application

IZO application is the Open Source type. It was developed in the free LAZARUS environment. The program consists of two main modules, which enable the calculation of coefficients and modeling of adsorption isotherms according to Freundlich, Langmuir, and BET based on experimental data and the prediction of the adsorption bed working time using formulas found in the literature.

#### 2.1.1. Coefficients of Adsorption Isotherms

**Figure 2:**
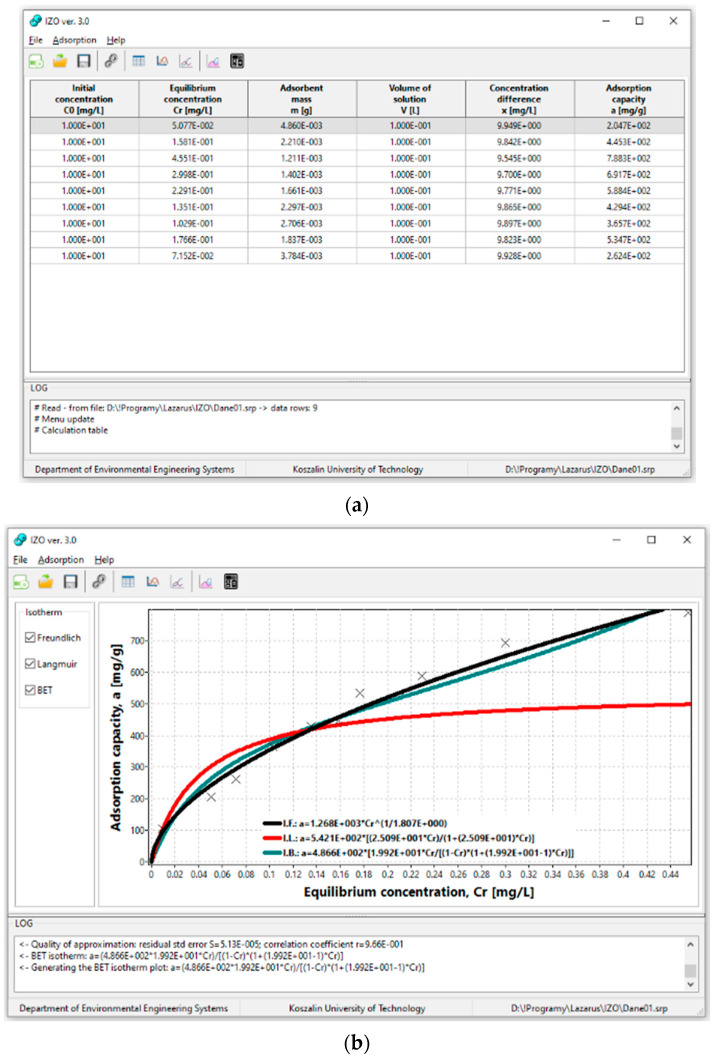
(**a**) Input of initial experimental data and (**b**) example graphs of adsorption isotherms according to Freundlich, Langmuir, and BET.

In the beginning, the app requires the introduction of independent variables as preliminary data in the form of a table (Figure 2a), i.e., initial concentration *C*_0_ [mg/dm^3^], equilibrium concentration *Cr* [mg/dm^3^], adsorbent mass *m* [g], and volume of the solution *V* [dm^3^]. The program controls whether the initial input data are correct and then calculates the resulting variable parameters in real mode, i.e., concentrations difference *x* [mg/dm^3^] and adsorption capacity *a* [mg/g]. In the case of incorrect values or lack of initial data, the IZO app does not calculate the resulting variables and does not take them into account during further calculations.

When at least three rows of data are input into the table, the app will generate a scatter plot of adsorption capacity *a* [mg/g] vs. the value of the equilibrium concentration *Cr* [mg/dm^3^] in module 1. Module 1 also calculates the coefficients of adsorption isotherms according to Freundlich, Langmuir, and BET and generates their graphs (Figure 2b). However, due to the specific form of the BET isotherm, there is the possibility of an asymptote occurrence. In such a case, coefficients of the BET isotherm cannot be calculated within the considered equilibrium concentration range. Therefore the IZO application generates a window with the information and does not present the plot of the BET isotherm.

The advantage of the IZO app for researchers is entering new points directly on the chart (Figure 2b). The point introduced on the graph is inserted into the table of initial data, and the app in real mode recalculates the values of adsorption isotherms coefficients (Figure 2a). In such a case, the app assumes the same values of initial concentration *C*_0_ and volume of solution V as in the table row preceding the new value. In the next step, based on the location of the new point on the chart, the app reads and enters into the table adsorption capacity a and equilibrium concentration *Cr*. Then it calculates the concentrations difference *x* and mass of adsorbent from the transformation *m* = *x*∙*V*∙*a*^−1^.

It is possible to simulate changes in the course of isotherms by changing the location of measurement points on the chart by moving them free-hand. The values of adsorption isotherms coefficients change in real mode. The change of location of the measurement points on the chart automatically adjusts the values of parameters in the initial data table (Figure 2a). If the point significantly deviates from the adsorption isotherms charts, it can be deleted. The app also updates the values of the coefficients of equations of adsorption isotherms.

**Figure 3:**
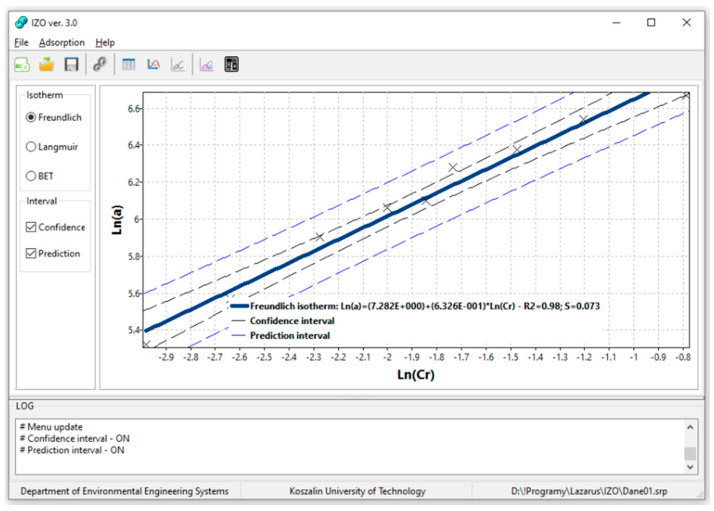
Visualization of an example linear plot of adsorption isotherms according to Freundlich.

The IZO app calculates the values of adsorption isotherms coefficients using the linearization method, as seen in Figure 3. That method transforms the isotherm equation so that the independent variable appears only once on the right side of the equal sign, and the coefficients are functions of constant parameters. On the left side of the equation, there are both independent and dependent variables. The linearization method makes it possible to verify the validity of theoretical assumptions—the experimental points should line up on a straight line in the transformed coordinate system. Moreover, it eliminates random errors (points deviating from the straight line) and allows the determination of constants included in the coefficients of the linearized equation.

In the case of Freundlich isotherm, the graph *a* = *x_m_*∙*Cr^n^* is transformed to the form Ln(*a*) = Ln(*b*_1_) *+ b*_2_∙Ln(*Cr*). Hence the values of the coefficients are *x_m_* = Exp(*b*_1_) and *n* = *b*_2_. The linearization of the Langmuir isotherm *a* = *x_m_*∙[(*K_L_*∙*Cr*)∙(1 *+ K_L_*∙*Cr*)^−1^] consists in the transformation to the form *a*^−1^ = *b*_1_ *+ b*_2_∙*Cr*^−1^, where the values of the coefficients are *x_m_* = *b*_1_^−1^, and *K_L_* = *b*_1_∙*b*_2_^−1^. The BET isotherm in the form *a* = *x_m_*∙(*K_B_*∙*Cr*)∙[(1 − *Cr*) (1 *+* (*K_B_* − 1) *Cr*)]^−1^ the program transforms to *Cr*∙[*a*∙(1 − *Cr*)]^−1^ = *b*_1_ *+ b*_2_∙*Cr*, hence the values of the coefficients are *x_m_* = (*b*_1_ *+ b*_2_)^−1^ and *K_B_* = 1 + *b*_2_∙*b*_1_^−1^. Coefficients *b*_1_ and *b*_2_ are calculated using linear least-squares approximation. The program uses the TFitSeries module from the Numlib library to calculate the quality of the approximation. It is determined using the least-squares method, which allows calculating the coefficient of determination *R*^2^, and the standard error of the regression *S*. The coefficient of determination *R*^2^ is a quotient of the sum of squared deviations and the sum of squared errors. Its value is describing how well the fitted curve explains the data variability. The *R*^2^ value is within the range of 0 to 1. *R*^2^ of 1 indicates that the regression predictions perfectly fit the data [21,26].

The application is also equipped with the function which presents lower and upper limits of confidence and prediction intervals for the fitted plot to analyze the data. Those limits determine the areas in which a fitted plot (confidence interval) or individual data points (prediction interval) can be expected based on a default probability value of 95%.

#### 2.1.2. Adsorption Bed Service Time

The 2nd module of the application calculates the duration of the adsorption process *t_S_* [h] in the adsorbent bed, based on three selected formulas found in the literature. The value of the adsorbate concentration in the adsorbent monolayer *x_m_* [g/kg] is the most important in those formulas. This parameter is calculated in module 1.

The first formula uses the relation resulting from the mass balance equation (MBE) transformation for the adsorbent layer. The formula uses the following parameters: height *H* [m], bulk density *g_w_* [kg/m^3^], initial concentration of medium *C*_0_ [kg/m^3^], assumed output concentration *Ce* [kg/m^3^], and flow speed *v_p_* [m/h]:

(1) $$t_{S}=\frac{x_{m}\cdot g_{w}\cdot H}{v_{p}\cdot \left(C_{0}-Ce\right)}$$

The duration of the adsorption process *t_S_* calculated using the balance Equation (1) transformation is longer than the actual service time of the sorbent bed. That is why, in practice, among others, the equation of Zuchowicki, Zabieziński, and Tichonow (ZZT) or equation of Bohart-Adams (BA) are used [21].

The ZZT equation derives from the kinetics of non-stationary external diffusion for the adsorption system described by the Langmuir equation, and Formula (2) expresses it.

(2) $$t_{S}=\frac{x_{m}\cdot g_{w}}{v_{p}\cdot C_{0}}\left\{H-v_{p}\cdot k_{e}^{-1}\left[w^{-1}\cdot \ln\left(C_{0}\cdot Ce-1\right)+\ln\left(C_{0}\cdot Ce\right)-1\right]\right\}$$

In the model derived from the equation of the adsorbate concentration field in the internal structure of the adsorbent, the value of the external mass transfer coefficient *ke* [s^−1^] is calculated based on the equivalent (averaged) diffusion coefficient *D_Z_* [m^2^/s], the porosity of the carbon layer *ε* [-], the radius of the intergrain capillary *r_K_* [m] and the active surface per unit volume of the bed *A_Z_* [m^2^/m^3^]:

(3) $$k_{E}=2\cdot D_{Z}\;\frac{A_{Z}}{r_{K}}\cdot \frac{1-\varepsilon }{\varepsilon }\;\left[\frac{1}{s}\right]$$

After simplifying Equation (3), the coefficient of external mass transfer *k_E_* [1/s] takes the form:

(4) $$k_{E}=144\cdot \frac{D_{Z}}{d_{M}^{2}}\cdot \frac{{\left(1-\varepsilon \right)}^{3}}{\varepsilon^{2}}\;\left[\frac{1}{s}\right]$$

Based on the value of the longitudinal diffusion coefficient *D_H_* [m^2^/s], the radius of the intergrain capillary *r_K_* [m], and the active surface per unit volume of the bed *A_Z_* [m^2^/m^3^], it is also possible to calculate the external mass transfer coefficient *k_E_* [m/s] of the adsorbate in the flow direction [9]:

(5) $$k_{E}=4\cdot \frac{D_{H}}{A_{Z}\cdot r_{K}^{2}}\;\left[\frac{m}{s}\right]$$

After simplifying Equation (5), the external mass transfer coefficient *k_E_* [m/s] takes the form:

(6) $$k_{E}=12\cdot \frac{D_{H}}{d_{M}}\cdot \frac{1-\varepsilon }{\varepsilon^{2}}\;\left[\frac{m}{s}\right]$$

The value of the external mass transfer coefficient *ke* [s^−1^] is calculated using the averaged diffusion coefficient *D* [m^2^/s], the porosity of the layer *ε_W_* [-], and the average grain sizes of the adsorbent *d* [mm]. The value of the parameter *w* [-] in Equation (2) is defined as the quotient of the initial concentration *C*_0_ to the concentration of the adsorbate in the stream equivalent to half the limit value of adsorption *C*_0.5_. Therefore, it is more convenient to use a simplified version of the ZZT equation in the form:

(7) $$t_{S}=\frac{x_{m}\cdot \rho_{N}}{v_{P}\cdot C_{0}}\left\{H-v_{p}\cdot k_{e}^{-1}\left[\ln\left(C_{0}\cdot Ce\right)-1\right]\right\}$$

BA Equation (8) lacks parameter *w* [-], compared to the ZZT (2) equation. As a result, Equation (8), similarly to Equation (7), refers to the adsorption isotherm only by the value of the concentration of the adsorbate in the monolayer *x_m_*.

(8) $$t_{S}=\frac{x_{m}\cdot g_{w}}{v_{p}\cdot C_{0}}\left\{H-v_{p}\cdot k_{e}^{-1}\left[\ln\left(C_{0}\cdot Ce-1\right)+\ln\left(C_{0}\cdot Ce\right)-1\right]\right\}$$

The lack of a parameter *w* in the Formula (8) does not significantly change the duration of the *t_S_* adsorption process. Like in Formula (7), the value of the first part of the Equations (2), (7) and (8), i.e., *x_m_·g_w_·*(*v_p_·C*_0_)^−1^ is much higher than the value of the rest of the equation concerning the difference in the height of the adsorbent bed and the adsorption front.

**Figure 4:**
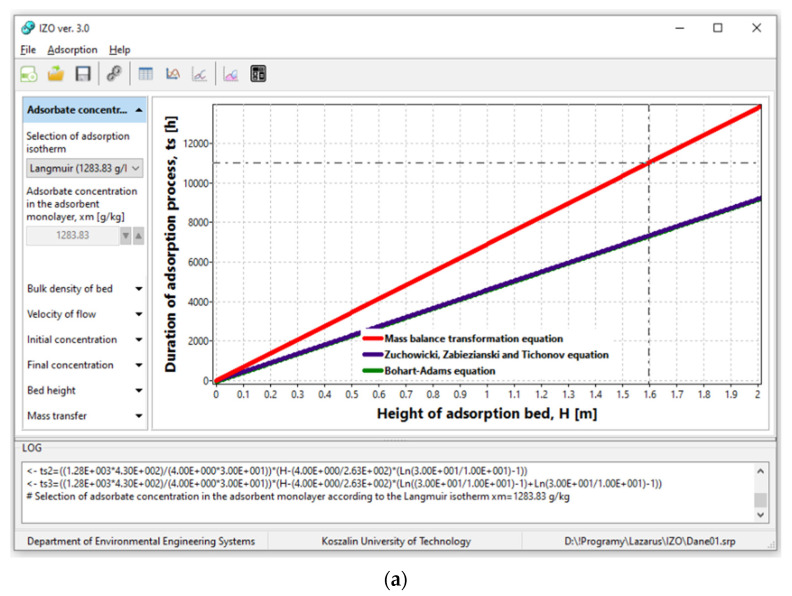

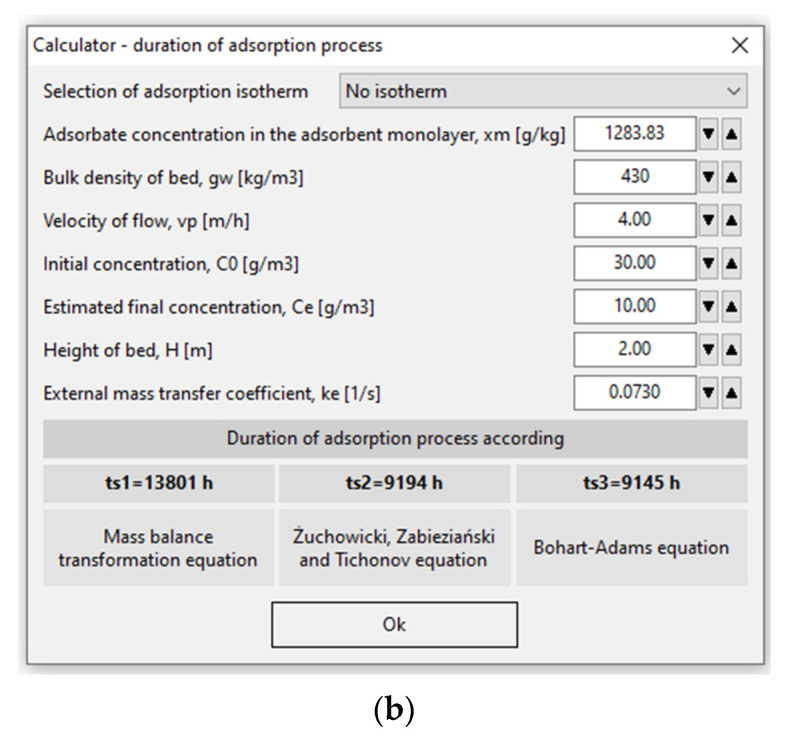
(**a**) An example graph of the duration of the adsorption process *t_S_* [h] vs. the height of the bed *H* [m] (**b**) and calculation of *t_S_* [h] depending on the change of selected independent variables.

The IZO application uses Equations (1), (7) and (8) to generate the graph of the duration of the adsorption process *t_S_* vs. the height of the adsorption bed *H* (Figure 4a). There is also a possibility to change the values of other independent parameters in limited ranges, i.e., the concentration of adsorbate in the monolayer *x_m_* (1 ≤ *x_m_* ≤ 2000) [g/kg], bulk density of the adsorbent *g_w_* (300 ≤ *g_w_* ≤ 500) [kg/m^3^], initial concentration *C*_0_ (0 ≤ *C*_0_ ≤ 10,000) [kg/m^3^], assumed final concentration *Ce* (0 ≤ *Ce* ≤ 10,000) [kg/m^3^], flow velocity *v_p_* (1 ≤ *v_p_* ≤ 20) [m/h], and external mass transfer coefficient *ke* (0.01 ≤*ke* ≤ 0.5) [s^−1^]. Besides, the application can import the calculated value of the adsorbate concentration in layer *x_m_* from module 1. However, the maximum time of the adsorption process *t_S_* during chart generation (Figure 4a) is calculated using Equation (1). In most cases, Equations (7) and (8) give similar results. That is why the lines representing mentioned equations overlap in Figure 4a.

An additional element in module 2 is the so-called calculator, which quickly calculates the duration of the adsorption process *t_S,_* taking into account changes of the mentioned parameters (Figure 4b). The calculator gives three answers according to Equations (1), (2) and (8).

The view of the graphs of adsorption isotherms (Figure 2b and Figure 3) and the duration of the adsorption process (Figure 4a) is fully adjustable. Moreover, all graphs may be copied to the system clipboard or saved as a graphic file (bmp, jpg, png).

**Figure 5:**
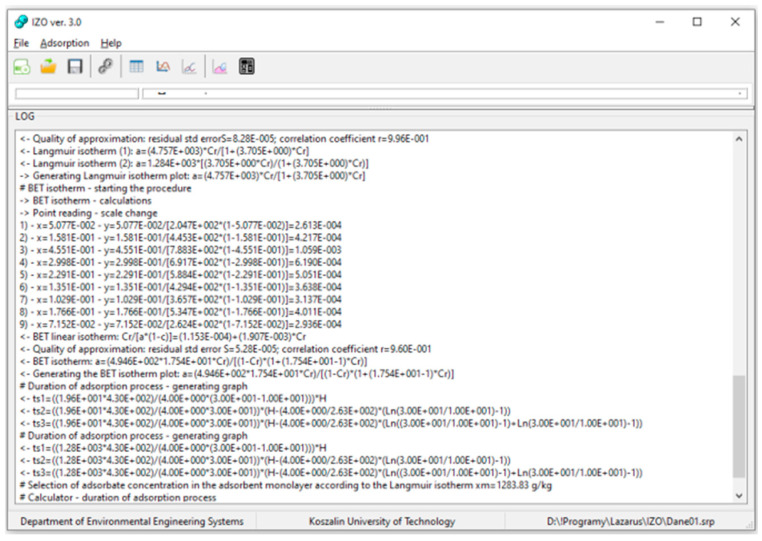
LOG window.

The application can also show a LOG window, where all operations carried out by the program are saved in real mode (Figure 5). The LOG window increases the transparency of all mathematical calculations performed.

Initial input data are saved as an external file in text format (*.srp). This file may be edited later in other external programs. The application also can generate a report containing a table with measurements (initial data) and the graph of adsorption isotherms. Besides, measurement data and results of calculations may be saved in external files in txt or pdf format to analyze them later in other applications.

### 2.2. Laboratory Adsorption Test

Laboratory adsorption tests were performed to check the correct functioning of the IZO application. The results of laboratory tests were entered into the app. The output of the calculations was the values of Freundlich, Langmuir, and BET isotherms. Simultaneously, using the same preliminary data, the coefficients of adsorption isotherms were calculated using nonlinear estimation in the Statistica program.

Laboratory adsorption tests were performed using the leachate from the municipal waste landfill. The adsorption process was carried out on selected activated carbons ORGANOSORB 10 (GLOBAL CONCEPTS 2000 POLSKA Sp. z o.o., Szczecin, Poland), DESOTEK (DESOREC ACTIVATED CARBON, Roeselare, Belgium), and BA-10 (Elbar–Katowice Sp z o.o., Racibórz, Poland). Tests were carried out in static conditions according to the methodology presented in [10]. This methodology enables a comparison of the adsorption capacity of adsorbates on different adsorbents.

The adsorbent was degassed, washed with distilled water, dried, and then placed in a spherical flask and dried to constant weight in an electric dryer at 150 °C for 3 h. Next, samples of prepared sorbent were added to the leachate. Containers with mixtures of leachate and adsorbent were first shaken manually to ensure complete wetting of the activated carbon and then mixed intensively in a FLOCCULATOR 5W6 (Carl ROTH, Karlsruhe, Germany) device at room temperature 20 °C ± 0.5 °C for 24 h. For the next 24 h, mixtures were left to reach adsorption equilibrium. Then, the samples were filtered twice using soft filter paper. The value of total organic carbon *TOC* [mg/dm^3^] was determined in the raw leachate and filtrates using the HACH DR/2010 spectrophotometer (HACH LANGE Sp. z o.o., Wrocław, Poland) [29].

During the test, a fixed initial concentration of TOC *C*_0_ = 504 mg/dm^3^ and a volume of solution *V* = 1.0 dm^3^ was used. The independent variable was the mass of adsorbent *m* [g], while the result variable was the equilibrium concentration *Cr* [mg/dm^3^].

## 3. Results

### Analysis of Calculation Results of Adsorption Isotherms Coefficients

The results of the tests of the static adsorption process of leachate from a municipal waste landfill are given in Table 1.

**Figure 6:**
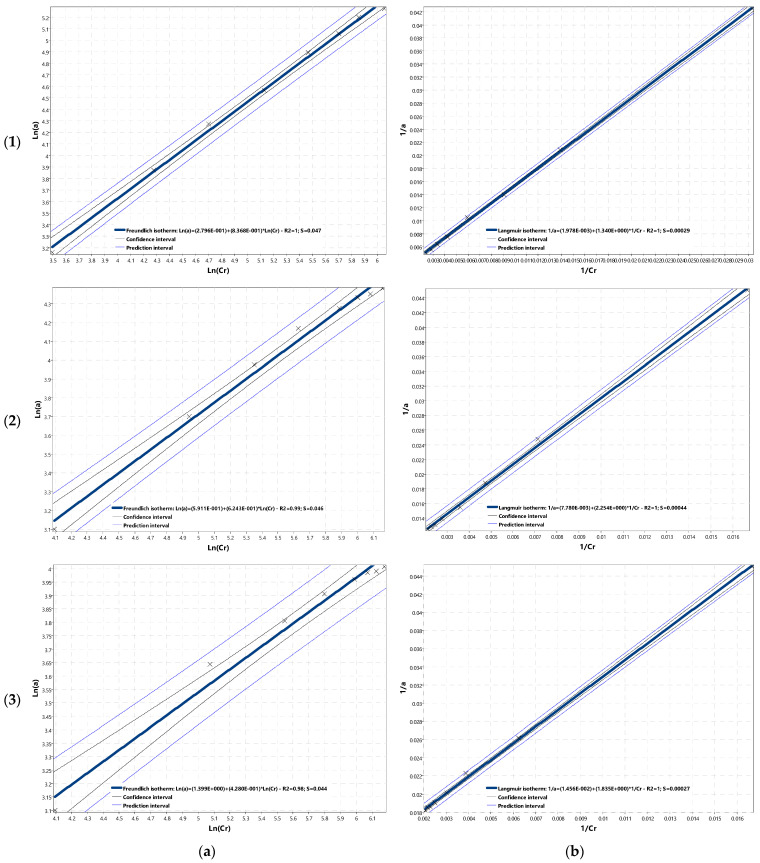
Charts of linear adsorption isotherms according to (**a**) Freundlich and (**b**) Langmuir for (**1**) ORGANOSORB 10, (**2**) DESOTEK, and (**3**) BA-10 carbons.

**Figure 7:**
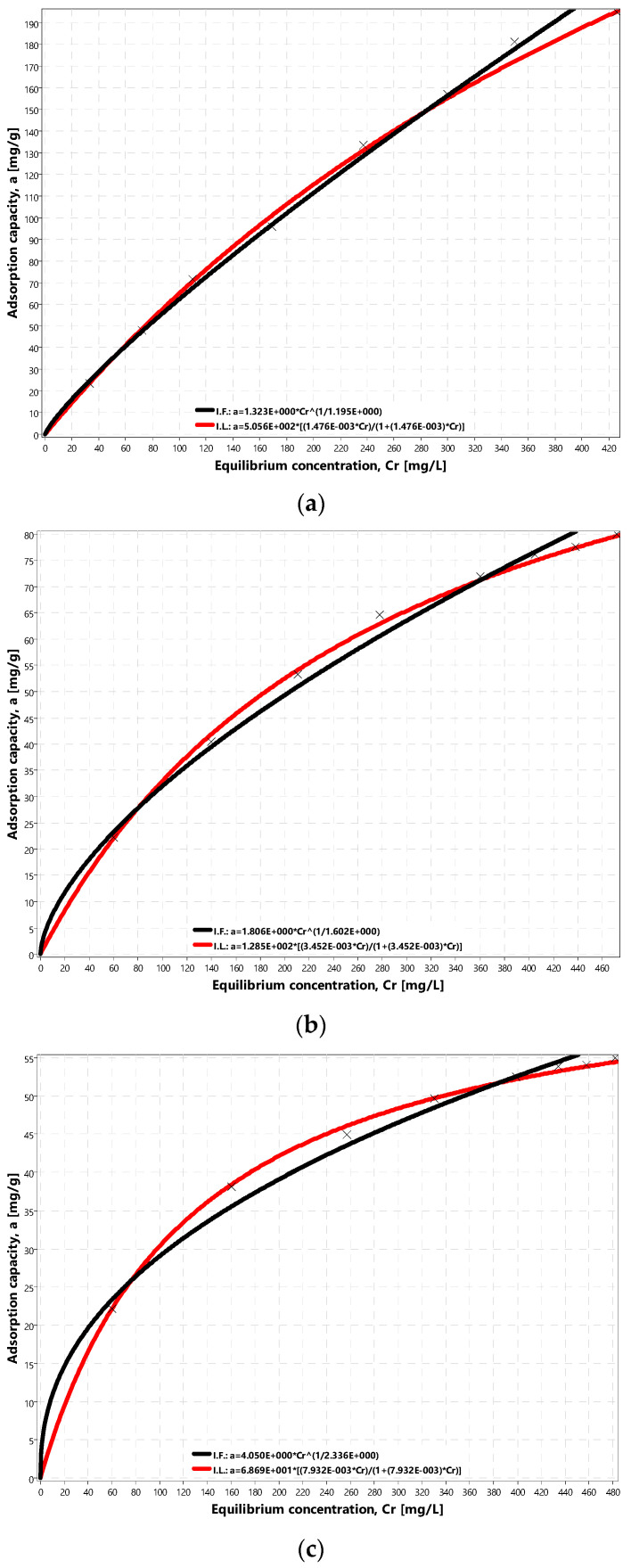
Charts of: (**a**) adsorption isotherms according to Freundlich and Langmuir for ORGANOSORB 10 carbon, (**b**) adsorption isotherms according to Freundlich and Langmuir for DESOTEK carbon, and (**c**) adsorption isotherms according to Freundlich and Langmuir for BA-10 carbon.

The values presented in Table 1 were introduced into the IZO application. Then, the program generated graphs and calculated the coefficients of adsorption isotherms, according to Freundlich and Langmuir, and generated graphs in linear form (Figure 6). In the case of the BET isotherm, coefficients cannot be calculated within the considered equilibrium concentration range because of the presence of asymptotes. Therefore the IZO application generates a window with the information and does not present the plot of the BET isotherm. Plots of adsorption isotherms according to Freundlich and Langmuir for considered adsorbents ORGANOSORB 10, DESOTEK, and BA-10, which the IZO application generates, are shown in Figure 7.

Simultaneously, the results of laboratory tests were introduced into the Statistica program, and the coefficients of adsorption isotherms were calculated using a nonlinear estimation method according to Gauss-Newton. The assumed convergence criterion was 1 × 10^−6^; the initial value for variables was 0.1. Table 2 presents a comparison of coefficients of adsorption isotherms calculated using a nonlinear estimation (Statistica) and linearization (IZO) along with a coefficient of determination *R*^2^ for each method.

Linearization and nonlinear calculation methods are comparable, based on the analysis of quality factors (Table 2).

The results of laboratory tests (presented in Table 1) are better described by isotherm according to Langmuir (Figure 7a–c). Graphs and calculated coefficients of fit quality (*R*^2^, *S*), presented in Figure 6, also confirm that. Better values of fit coefficients for Langmuir isotherm may also prove that within the range of values parameters applied during the experiment, monolayer adsorption took place. ORGANOSORB 10 proved to be the best adsorbent. The concentration of adsorbate in the monolayer of ORGANOSORB 10 adsorbent, calculated using the linearization method, was 506 g/kg and using nonlinear estimation, was 552 g/kg.

**Figure 8:**
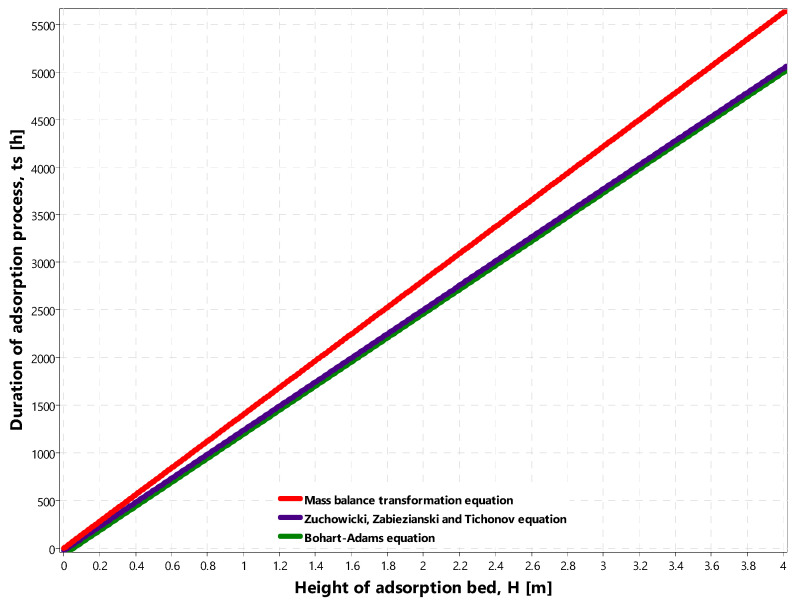
Duration of the adsorption process *t_S_* [h] vs. the height of the adsorption bed *H* [m] calculated using the transformation of the mass balance equation (MBE), the Zuchowicki, Zabieziński, Tichonow (ZZT), and the Bohart-Adams (BA) equations.

Figure 8 presents the duration of adsorption process *t_S_* along with the increase in adsorption bed height *H* (for ORGANOSORB 10) within the range 0 to 4 m. The calculations were conducted using the following parameters:

- the adsorbate concentration in the adsorbent monolayer *x_m_* = 506 g/kg;
- bulk density of the ORGANOSORB 10 adsorbent *g_w_* = 500 kg/m^3^;
- the initial concentration of TOC in the wastewater *C*_0_ = 50 mg/dm^3^;
- assumed final concentration of TOC *Ce* = 5 mg/dm^3^;
- flow rate *v_p_* = 4 m/h;
- external mass transfer coefficient *ke* = 0.073 1/s.

The calculated duration of the adsorption process changes in the range 0 to 6325 h for MBE, while for the ZZT equation, it is in the range 0 to 5048 h, and for the BA equation, 0 to 5022 h (Figure 8).

## 4. Discussion

The interpretation of adsorption isotherms is mostly dependent on correct modeling, which is currently conducted using complex mathematical computer programs or software environments. These applications force on user knowledge of complicated operations or even programming. The IZO application presented in this paper is a specialist supplement to this issue. It enables the calculation of coefficients and visualization of adsorption isotherms according to Freundlich, Langmuir, and BET in a classic and linear system, in a simple communicative way. The application also allows users to calculate the working time of the adsorption bed based on the transformation of the mass balance equation, and according to the Zuchowicki, Zabieziński, Tichonow, and the Bohart-Adams equations.

An excellent aid in research work is changing the location and simultaneously editing values of measurement points directly on the adsorption isotherms chart. This way, users may observe real-time changes in equations and graphs, particularly the value of adsorbate concentration in the adsorbent monolayer *x_m_*.

The results of tests conducted in actual conditions confirm that the linearization method of the calculation of adsorption isotherms coefficients, used in the IZO application, gives sufficient accuracy and may be used as an alternative of, e.g., the nonlinear estimation method. However, users must consider the fact that after the linearization of the equation, the real accuracy of the fit coefficients is no longer obtained. The linearization of the adsorption equation is an acceptable method of obtaining preliminary confirmation that the data are consistent with the assumptions of the first-order Langmuir model. Due to the specific form of the BET isotherm, there is the possibility of an asymptote occurrence. Future versions of the IZO application will solve this problem by, for example, changing the equilibrium concentration value and expressing it in g/dm^3^.

The IZO application is an Open Source program. It is constantly developed and is available for free both in the form of source code and compiled version on the website https://app.ros.edu.pl (accesed on 21 July 2021). Users are free to develop it further and verify its functioning. Users can also add or modify existing functions to suit their specific needs. The source code can be compiled for any system environment (Windows, Linux, or Mac OS X).

## Figures and Tables

**Table 1:** Impact of adsorbent dose *m* [g] on the value of the equilibrium concentration *Cr* [mg/dm^3^] in the process of static adsorption of leachate from municipal landfill on ORGANOSORB 10, DESOTEK, and BA-10 activated carbons.

Parameter	Sample No.							
	1	2	3	4	5	6	7	8
Adsorbent dose, m [g]	20	9.0	5.5	3.5	2.0	1.3	0.85	0.40
Equilibrium concentration, *Cr* [mg/dm^3^]								
ORGANOSORB 10	33	72	110	169	237	300	350	426
DESOTEK	60	140	211	278	360	405	438	472
BA-10	68	160	257	330	399	434	458	482

**Table 2:** Comparison of linearization and nonlinear estimation methods of calculation of adsorption isotherms coefficients.

No.	Adsorbent	Method of Calculation	Coefficients of Adsorption Isotherms			
			Freundlich		Langmuir	
			*x_m_*	*n*	*x_m_*	*K_L_*
1	ORGANOSORB 10	Linearization	1.32	1.20	505.60	1.48 × 10^−3^
		*R* ^2^	1.00		1.00	
		Non-linear estimation	1.80	1.28	551.88	1.33 × 10^−3^
		*R* ^2^	0.99		0.99	
2	DESOTEK	Linearization	1.81	1.60	128.50	3.45 × 10^−3^
		*R* ^2^	0.99		1.00	
		Non-linear estimation	2.41	1.75	133.82	3.21 × 10^−3^
		*R* ^2^	0.99		1.00	
3	BA-10	Linearization	4.05	2.34	68.69	7.93 × 10^−3^
		*R* ^2^	0.98		1.00	
		Non-linear estimation	4.88	2.52	69.88	7.50 × 10^−3^
		*R* ^2^	0.99		0.99	

## Data Availability

Not applicable.

## References

[1] Ruthven D.M. (2020). Principles of Adsorption and Adsorptionprocess.

[2] Singh J.K., Verma N. (2020). Aqueous Phase Adsorption Theory, Simulations and Experiments.

[3] Kuśmierek K., Bieniek K., Dąbek L., Świątkowski A. (2017). Adsorption of Halogenophenols from Aqueous Solutions on Activated Carbon. Ann. Set Environ. Prot..

[4] Petkovska M. (2014). Discrimination between Adsorption Isotherm Models Based on Nonlinear Frequency Response Results. Adsorption.

[5] Rouquerol J. (2013). Adsorption by Powders and Porous Solids.

[6] Worch E. (2012). Adsorption Technology in Water Treatment. Fundamentals, Processes, and Modeling.

[7] Saadi R., Saadi Z., Fazaeli R., Fard N.E. (2015). Monolayer and Multilayer Adsorption Isotherm Models for Sorption from Aqueous Media. Korean J. Chem. Eng..

[8] Tóth J. (2002). Adsorption: Theory, Modeling, and Analysis.

[9] Duong D.D. (1997). Adsorption Analysis: Equilibria and Kinetics.

[10] Ignatowicz K., Piekarski J., Skoczko I., Piekutin J. (2016). Analysis of Simplified Equations of Adsorption Dynamics of HCH. Desalin. Water Treat..

[11] Freundlich H. (1907). Über Die Adsorption in Lösungen. Z. Phys. Chem..

[12] Yanniotis S., Blahovec J. (2009). Model Analysis of Sorption Isotherms. LWT Food Sci. Technol..

[13] Ghaedi M. (2021). Adsorption: Fundamental Processes and Applications.

[14] Langmuir I. (1916). The constitution and fundamental properties of solids and liquids. Part I. solids. J. Am. Chem. Soc..

[15] Langmuir I. (1917). The constitution and fundamental properties of solids and liquids. Part II. liquids. J. Am. Chem. Soc..

[16] Brunauer S., Emmett P.H., Teller E. (1938). Adsorption of Gases in Multimolecular Layers. J. Am. Chem. Soc..

[17] Heraldy E., Hidayat Y., Firdaus M. (2016). The Langmuir Isotherm Adsorption Equation: The Monolayer Approach. IOP Conf. Ser. Mater. Sci. Eng..

[18] Piekarski J. (2007). Numeric simulation of selected parameters of the sorption process. Pol. J. Environ. Stud..

[19] Ruthven D.M. (2000). The Rectangular Isotherm Model for Adsorption Kinetics. Adsorption.

[20] Chen X. (2015). Modeling of Experimental Adsorption Isotherm Data. Information.

[21] Piekarski J. (2009). Numeric Modelling of Filtration and Sorption Process.

[22] Patel H. (2019). Fixed-Bed Column Adsorption Study: A Comprehensive Review. Appl. Water Sci..

[23] Piekarski J., Dąbrowski T. (2009). Numerical Method of Assessing the Sorption of Pollutants from Wastewater. Miner. Resour. Manag..

[24] Kumar K.V., Ramamurthi V., Sivanesan S. (2005). Modeling the Mechanism Involved during the Sorption of Methylene Blue onto Fly Ash. J. Colloid Interface Sci..

[25] Golovko O., de Brito Anton L., Cascone C., Ahrens L., Lavonen E., Köhler S.J. (2020). Sorption Characteristics and Removal Efficiency of Organic Micropollutants in Drinking Water Using Granular Activated Carbon (GAC) in Pilot-Scale and Full-Scale Tests. Water.

[26] Subramanyam B., Das A. (2014). Linearised and Non-Linearised Isotherm Models Optimization Analysis by Error Functions and Statistical Means. J. Environ. Health Sci. Eng..

[27] Agarwal A.K., Kadu M.S., Pandhurnekar C.P., Muthreja I.L. (2016). Brunauer-Emmett-Teller (B.E.T.), Langmuir and Freundlich Isotherm Studies for the Adsorption of Nickel Ions onto Coal Fly Ash. AJW.

[28] Kyzioł-Komosińska J., Rosik-Dulewska C., Pająk M., Czupioł J., Dzieniszewska A., Krzyżewska I. (2015). Sorption of Acid Green 16 from Aqueous Solution onto Low-Moor Peat and Smectite Clay Co-Occurring in Lignite of Belchatow Mine Field. Ann. Set Environ. Prot..

[29] Puchlik M., Ignatowicz K., Dabrowski W. (2015). Influence of bio-preparation on wastewater purification process in constructed wetlands. J. Ecol. Eng..

